# Genomic Risk Models Improve Prediction of Longitudinal Lipid Levels in Children and Young Adults

**DOI:** 10.3389/fgene.2013.00086

**Published:** 2013-05-21

**Authors:** Nathan E. Wineinger, Andrew Harper, Ondrej Libiger, Sathanur R. Srinivasan, Wei Chen, Gerald S. Berenson, Nicholas J. Schork

**Affiliations:** ^1^Scripps Translational Science InstituteLa Jolla, CA, USA; ^2^Newcastle UniversityNewcastle upon Tyne, Tyne and Wear, UK; ^3^Scripps Research InstituteLa Jolla, CA, USA; ^4^Center for Cardiovascular Health, Tulane UniversityNew Orleans, LA, USA

**Keywords:** lipids, polygenic model, prediction, cardiovascular diseases, statistical methods

## Abstract

In clinical medicine, lipids are commonly measured biomarkers used to assess an individual’s risk for cardiovascular disease, heart attack, and stroke. Accurately predicting longitudinal lipid levels based on genomic information can inform therapeutic practices and decrease cardiovascular risk by identifying high-risk patients prior to onset. Using genotyped and imputed genetic data from 523 unrelated Caucasian Americans from the Bogalusa Heart Study, surveyed on 4,026 occasions from 4 to 48 years of age, we generated various lipid genomic risk models based on previously reported markers. We observed a significant improvement in prediction over non-genetic risk models in high density lipoprotein cholesterol (increase in the squared correlation between observed and predicted values, Δ*R*^2^ = 0.032), low density lipoprotein cholesterol (Δ*R*^2^ = 0.053), total cholesterol (Δ*R*^2^ = 0.043), and triglycerides (Δ*R*^2^ = 0.031). Many of our approaches are based on an *n*-fold cross-validation procedure that are, by design, adaptable to a clinical environment.

## Author Summary

Genomic studies have produced promising results which have guided researchers studying the etiology of complex disease. Yet, the transition from bench to bedside has been less successful for a variety of reasons, including the lack of clinically relevant results and the failure to replicate across studies. We provide a framework for applying previously reported genomic results to lipid level prediction by leveraging a polygenic model. Our designs are based on *n*-fold cross-validation, which naturally mimic a dynamic, sequentially updated clinical environment. We expect variations of our approach may be implemented in future clinical applications as patients’ genomic information becomes more readily available and medicine moves toward personalized risk assessment.

## Introduction

Cardiovascular disease is a leading cause of mortality and morbidity throughout the urbanized world, largely due to atherosclerosis (National Heart, 2012). Risk models designed to predict cardiovascular disease have been generated using traditional risk factors (Siontis et al., 2012). Lipids, specifically high density lipoprotein cholesterol (HDL-C), low density lipoprotein cholesterol (LDL-C), total cholesterol (TC), and triglycerides (TG) are among the most commonly measured biomarkers within clinical medicine, used to screen and assess individual risk for cardiovascular diseases. A surfeit of common variants implicated in lipid metabolism has been discovered through genome-wide association studies (GWAS) (Hindorff et al., 2009). Yet, in clinical practice the heritable component of cardiovascular disease risk has been largely neglected as the utility, practicality, and strategy for incorporating these results remains uncertain.

Biases in publishing are well-documented (Munafò et al., 2004; Dwan et al., 2008), and genetic results often fail to replicate across studies and/or populations (Greene et al., 2009; Dumitrescu et al., 2011). Even in the absence of missing heritability (Manolio et al., 2009), these present challenges in the transition from bench to bedside. One method to circumvent this problem is to treat the patient population as a continually growing sample from which one can fit models, draw inferences, and make predictions. When updated sequentially, such data can be used to evaluate future patients against those previously admitted based on relevant factors. To an extent, this practice is routinely performed in a clinical setting where a physician assesses risk based on published findings on non-genetic factors and prior experience. However, implementing genomic information requires a more refined approach as the amount of data is vast and the effect of any individual genetic marker is likely small (Park et al., 2010, 2011).

There are various methods designed to integrate the results from genetic association studies into individual prediction. Machine learning approaches are becoming more common, but require a more complex interpretation (Wei et al., 2009; Okser et al., 2010). Allele-counting methods (Lango et al., 2008; Evans et al., 2009) and its extension to weighted allele-counting methods (International Schizophrenia Consortium et al., 2009; Paynter et al., 2010) are popular and easily implemented. The latter contains a more natural interpretation of genomic risk but assumes genetic predictors are measured accurately. We assessed the predictive performance of various polygenic models on lipid levels in a longitudinal cohort, from adolescence to middle life, of unrelated European Americans from the Bogalusa Heart Study (BHS) (Frerichs et al., 1976). We found that these approaches can substantially improve prediction but appear to be limited to methods involving only markers previously reported to be associated with the phenotype.

## Results

The BHS is a long-term, longitudinal cohort study investigating cardiovascular disease progression in children in and around Bogalusa, Louisiana. Among the study participants with available genetic data, we identified a sample of 523 individuals, surveyed at age 4–48 years old on 4,026 occasions, that were genetically determined to be ancestrally European and unrelated to other sample participants (see Materials and Methods). Sample statistics are presented in Table 1.

**Table 1:** **Sample summary statistics (*n* = 523)**.

	Mean (SD)
Age at first observation (years)	10.2 (3.4)
Number of observations	7.7 (1.9)
Years between observations*	3.0 (3.5)
HDL cholesterol (mg/dL)	52.3 (18.2)
LDL cholesterol (mg/dL)	108.2 (35.4)
Total serum cholesterol (mg/dL)	174.8 (38.7)
Serum triglycerides (mg/dL)*	82.0 (62.0)
Systolic blood pressure (mmHg)	109.2 (11.9)
Diastolic blood pressure (mmHg)	70.2 (10.3)

*Indicates median (interquartile range).

**Figure 1:**
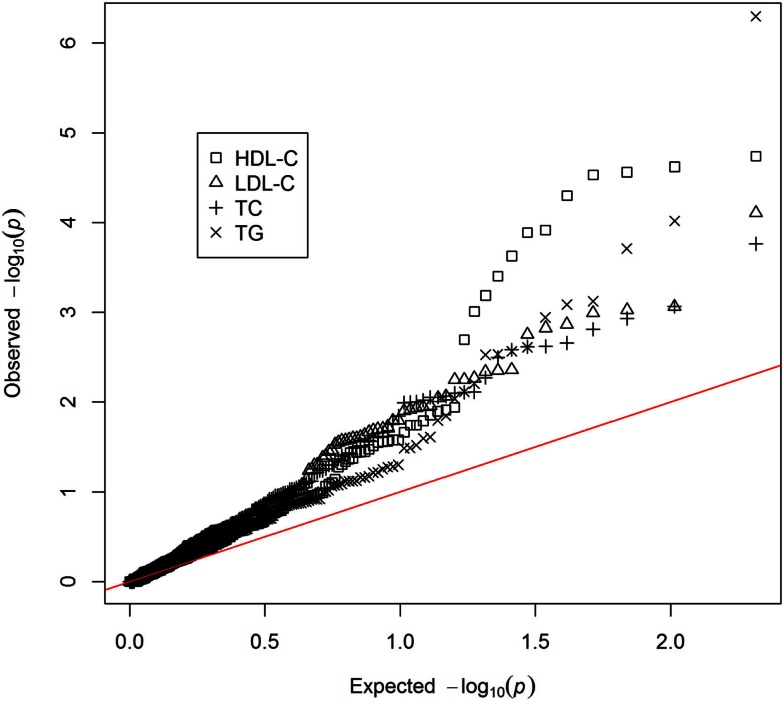
**QQ-plot of single marker associations between previously reported genetic risk markers and lipid levels**.

**Figure 2:**
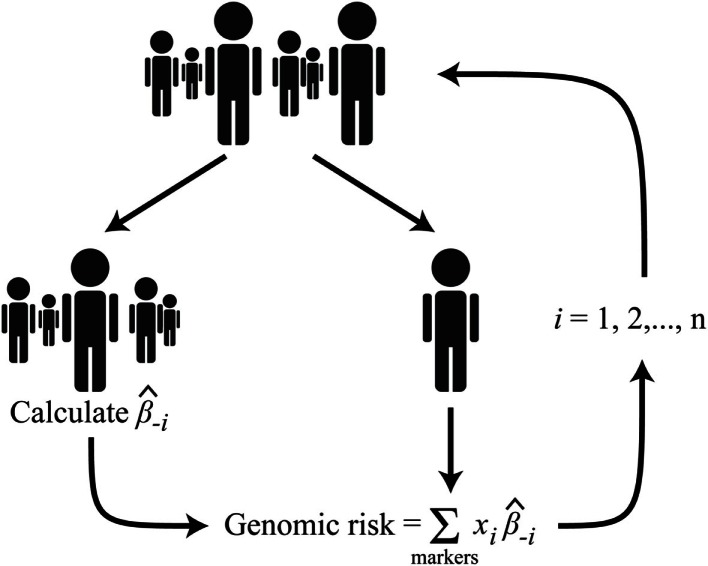
**Diagram of the *n*-fold cross-validation approach used to calculate the BHS genomic risk scores; where *i* is the index corresponding to the *i*th subject, ${\hat{\beta }}_{-i}$ is the marker effect calculated after excluding the *i*th subject, and *x_i_* is the genotype (additive coding) of the *i*th subject**.

Genetic markers previously reported to be associated with HDL-C, LDL-C, TC, or TG were extracted (Dataset S1 in Supplementary Material) – including genotyped (*n* = 117) and imputed markers (*n* = 90). Of these, 77 were previously reported to be associated with HDL-C, 62 with LDL-C, 65 with TC, and 40 with TG. Single marker association analyses were performed on all the extracted genetic markers for each lipid phenotype (Dataset S2 in Supplementary Material). Though no individual marker reached the threshold for genome-wide significance, there was a non-uniform distribution of *p*-values with a trend toward association (Figure 1). This provided an indication that polygenic models could explain a reasonable portion of the variation in lipid levels, above and beyond traditional, non-genetic risk factors. Seven genomic risk models were calculated, and are referred to as the NCBI risk score, BHS-A and BHS-R risk scores, BLR-A and BLR-R, and BRR-A and BRR-R. Each method is described in the Methods and a general summary of the *n*-fold cross-validation approach implemented in the BHS scores is presented in Figure 2.

The agreement between observed and predicted outcomes for each risk score are presented in Table 2. The predictive accuracy was measured as the square of the Pearson’s correlation between the observed and predicted outcomes (and is referred to as *R*^2^ in the remainder of the text). Non-genetic models were first considered, which included only the covariates age, sex, and body mass index. For these risk models, *R*^2^ ranged from 0.137 for HDL-C to 0.223 for LDL-C. These values were 0.183 and 0.218 for TC and TG, respectively.

**Table 2:** **Predictive accuracy, measured as the square of the Pearson’s correlation between observed and predicted outcomes**.

	HDL-C	LDL-C	TC	TG
Non-genetic	0.137 (0.119, 0.156)	0.223 (0.201, 0.248)	0.183 (0.161, 0.205)	0.218 (0.189, 0.249)
NCBI risk score	0.154 (0.133, 0.175)	0.276 (0.249, 0.302)	0.226 (0.202, 0.252)	0.249 (0.220, 0.280)
BHS-A risk score	0.152 (0.133, 0.172)	0.254 (0.228, 0.280)	0.211 (0.188, 0.237)	0.227 (0.199, 0.260)
BHS-R risk score	0.156 (0.136, 0.178)	0.262 (0.237, 0.288)	0.215 (0.191, 0.240)	0.238 (0.210, 0.270)
BLR-A	0.161 (0.143, 0.182)	0.258 (0.233, 0.285)	0.210 (0.185, 0.234)	0.234 (0.202, 0.267)
BLR-R	0.169 (0.148, 0.190)	0.258 (0.233, 0.283)	0.211 (0.188, 0.235)	0.236 (0.205, 0.269)
BRR-A	0.158 (0.139, 0.179)	0.259 (0.233, 0.286)	0.210 (0.187, 0.236)	0.230 (0.200, 0.262)
BRR-R	0.167 (0.147, 0.189)	0.257 (0.232, 0.283)	0.211 (0.188, 0.236)	0.235 (0.205, 0.269)

*Percentile bootstrap 95% confidence intervals based on 2,000 replicates are included in parentheses*.

The NCBI risk score was constructed using only markers previously reported to be associated with each lipid phenotype. It is identical to the weighted allele-counting method, where each weighting factor was assigned its previously reported estimate. The inclusion of the NCBI risk scores statistically improved predictive accuracy for each lipid phenotype beyond the non-genetic models. The largest improvement in *R*^2^ occurred for LDL-C which increased 0.052 to 0.276 (*p* = 2.32 × 10^−15^). TC increased 0.043 (*p* = 1.04 × 10^−13^), TG increased 0.031 (*p* = 9.58 × 10^−13^), and HDL-C increased 0.017 (*p* = 4.26 × 10^−8^).

The structure of the BHS-A and BHS-R risk scores are similar to the weighted allele-counting method, but with weights assigned for each individual based on estimates calculated from the remaining study participants – an implementation of *n*-fold cross-validation. BHS-A scores were calculated using all markers previously reported to be associated with HDL-C, LDL-C, TC, or TG. BHS-R scores, like the NCBI risk scores, were calculated using only markers that were previously found to be associated with the particular lipid phenotype. We use the letter A to refer the entire set of All previously reported lipid risk markers and R to refer to the set of markers previously Reported with each lipid (see Dataset S1 in Supplementary Material for a comprehensive marker list). For each phenotype, the inclusion of either risk score statistically improved *R*^2^. The BHS-R risk scores performed better than the BHS-A scores. This can likely be attributed to an increase in variance introduced in the BHS-A scores by including a larger portion of non-causal markers. Considerable improvements in *R*^2^ were achieved using the BHS-R risk scores over the non-genetic models. The largest absolute increase occurred with LDL-C rising 0.039 over the non-genetic model (*p* = 6.53 × 10^−12^). TC had a similar improvement (0.032, *p* = 3.76 × 10^−11^), while both HDL-C and TG had smaller, yet moderate improvements (0.019, *p* = 7.20 × 10^−9^; and 0.020, *p* = 6.14 × 10^−8^, respectively).

The Bayesian lasso regression (BLR) and Bayesian ridge regression (BRR) models fit each marker simultaneously using a Bayesian lasso and ridge penalty, respectively. Among all models, the BLR-R model was best at predicting HDL-C levels, increasing *R*^2^ 0.032 over the non-genetic model. These models performed on par with the NCBI and BHS risk scores (Table 2). The BLR and BRR models are better designed to detect novel, predictive loci as larger numbers of likely non-causal markers can be included in the polygenic model without significantly decreasing the predictive accuracy. This is shown by comparing the difference in *R*^2^ between BLR-R and BLR-A (and BRR-R and BRR-A) against the difference between the BHS-R and BHS-A – the former which tend to be smaller. As the decrease in *R*^2^ in the BHS models appears to be non-trivial when including a larger proportion of markers not previously associated, one should be cautious of recent findings (Chatterjee et al., 2013; Dudbridge, 2013) which advocate a more liberal threshold for the inclusion of genetic variants in polygenic prediction models.

Finally, the NCBI risk score model was applied to select age ranges to assess the predictive performance of the genetic risk at different time points, from childhood into adulthood, against a non-genetic model (Table 3). Significance and *R*^2^ was calculated at 10 years age intervals from 10 to 40 years of age after controlling, and not controlling for the individual’s previous lipid measurement. Across age ranges, the NCBI risk scores were significantly associated with the outcomes when previous lipid levels were not considered. In general, there did not appear to be a trend toward increased to decreased prediction across time. The largest gains in prediction over the non-genetic model occurred at age 30 for HDL-C and age 20 for the other lipid measurements. Alternatively, controlling for an individual’s previous lipid measurement tended to decrease the gain in predictive accuracy of the NCBI risk model – in a number of instances resulting in no association between the risk score and the outcomes. These results suggest that the combined influence of known genetic risk of lipid levels is substantial and roughly stable over time; but only in certain cases (e.g., TG at age 20) do known genetic risk estimates provide inference above and beyond the individual’s lipid history – otherwise little may be gained.

**Table 3:** **Predictive accuracy of the NCBI risk score for age-specific lipid levels**.

Age	Previous measurement?	HDL-C		LDL-C		TC		TG	
		Δ*R*^2^	*p*	Δ*R*^2^	*p*	Δ*R*^2^	*p*	Δ*R*^2^	*p*
10	No	0.025	8.63 × 10^−4^	0.060	9.71 × 10^−7^	0.049	3.30 × 10^−6^	0.010	0.016
20		0.018	5.49 × 10^−3^	0.074	5.32 × 10^−8^	0.088	3.05 × 10^−9^	0.055	8.75 × 10^−6^
30		0.037	7.15 × 10^−5^	0.063	7.60 × 10^−7^	0.044	2.96 × 10^−5^	0.026	4.28 × 10^−4^
40		0.017	3.91 × 10^−3^	0.037	2.47 × 10^−4^	0.044	5.38 × 10^−5^	0.048	3.49 × 10^−5^
20	Yes	–	0.116	0.013	1.31 × 10^−3^	0.022	8.12 × 10^−5^	0.064	4.98 × 10^−6^
30		0.025	3.80 × 10^−4^	0.008	0.016	–	0.092	0.009	0.023
40		–	0.740	–	0.503	0.006	0.039	0.025	8.63 × 10^−4^

*Δ*R*^2^ represents the increase in *R*^2^ over a non-genetic model. The model was assessed when the previous lipid measurement was not controlled for as a covariate (No), and when it was (Yes)*.

## Discussion

Risk prediction models have been successfully utilized in cardiovascular medicine to enable appropriate risk stratification of patients. Leveraging genomic data to further enhance cardiovascular risk prediction is an attractive option as many traditionally low-risk individuals routinely experience cardiovascular events, potentially explained by genomic susceptibility. While the models we propose provide substantial improvements over the non-genomic models, our models focus on intermediate phenotypes (i.e., lipids) associated with cardiovascular diseases rather than cardiovascular diseases themselves. Until further follow-up data incorporates clinical end-points, specifically heart attack, stroke, and death, our results, if used in clinical or public health contexts, would only bear on raising an individual’s awareness of an increased or decreased risk of lipid levels. This is not to say that genomic testing should be performed as a substitute for lipid measurements. Clearly if an individual is able to provide a DNA sample at some point in time, they are also likely to be able to provide a blood sample for lipid measurement at that time. Rather, in the presence of available genomic data (e.g., if or when sequencing is routinely performed on all patients), genomic risk metrics like the models we proposed may be used to guide treatment, inform interventions, or identify high-risk patients prior to onset.

The benefits of implementing genomic approaches into personalized health care have become a popular topic as the medical community appears ripe to integrate decades’ worth of scientific evidence into clinical practice. We expect the attraction of genotyping will continue to grow as sequencing costs steadily decline. However, there are looming questions concerning genomics’ usefulness in the clinic and the manner in which it is applied. We have demonstrated a number of methods for predicting lipid levels, based on previously reported genetic findings, which provide an improvement over non-genetic models. While the NCBI risk scores tended to perform slightly better – likely as a result of the individual estimates originating from larger studies and leading to less uncertainty in the parameters – we highlight the utility of the BHS, BLR, and BRR as estimates obtained from these methods are calculated within the study cohort; thus, potentially eliminating variation between studies and/or populations. Meanwhile, researchers or clinicians constructing polygenic risk scores generated from publicly available data, such as the traditional weighted allele-counting method, should be cautious. From an initial set of 271 markers we identified in the GWAS Catalog^1^ as being previously reported to be associated with lipid levels, we discovered that 38 referenced a different (incorrect) marker than the original study, and the correct marker could not be determined in three additional cases after reviewing the manuscripts. The interpretation of the parameters estimates also present some challenges. The most commonly used lipid units reported in previous studies, and those presented here, are in mg/dL. However, in some instances estimates were presented in mmol/L, scaled to the phenotypic variance, or both. We successfully converted 55 such markers, but could not unambiguously determine the parameters estimates on 21 other occasions.

The *n*-fold cross-validation approach we implemented in this study mimics a dynamic clinical setting where records are maintained on previously admitted patients, and a subsequent patient may be evaluated based on how he or she likens to these others. It is illogical to believe risk estimates should remain fixed over time and additional data should be ignored. Instead, risk models can be continually updated as more patient outcomes are observed and the predictive accuracy of the training dataset improves. However, how to best construct such a sequentially updated database for group specific prediction is unclear, and requires far more attention than we were able to provide in this study. How should the database initially be constructed to ensure stability in the predictions for the first set of patients? How should the predictive model be designed to account for emerging predictors or effects which change over time? What effect will un-blinding the patient or physician, by providing prediction results, have on intervention outcomes and future predictions? These questions, and others, must first be addressed.

There are options which may have improved our approach. First, as part of the cross-validation, we chose to exclude all observations from a study participant prior to assigning genomic risk and evaluating prediction. We could have improved our overall accuracy by only excluding the observation (and later observations) that we were attempting to predict. For example, if our intention was to predict a participant’s LDL-C at age 30, we could have included the participant’s observations before age 30 and excluded the others. This would equate to estimating and subsequently incorporating the random effect corresponding to each participant at each observation. There are challenges in properly summarizing these results as the accuracy would be greater in participants with more observations. We also note that the participant’s genomic risk score would differ at each time point. Though such an analysis is more computationally demanding, in practice we expect this approach to be ideal. If variations of these approaches were to be used in clinical practice, risk estimates would need to be recalculated for each newly admitted patient (or observation). Bayesian approaches, like the BLR and BRR, which provide a more straight-forward method of updating results with the acquisition of new data, would be more reasonable in this setting. Finally, we did not account for linkage disequilibrium between previously reported lipid genetic risk markers or model gene-gene and gene-environment interactions. Properly modeling linkage disequilibrium could improve prediction. Meanwhile, the role of interactions (Zuk et al., 2011) and their impact on prediction is uncertain (Aschard et al., 2012). Yet, we expect that the approaches presented here could be adapted to accommodate such effects.

The results from GWAS and the ubiquitous nature of missing heritability have proven that polygenic methods must be utilized in order to garner any clinically relevant, actionable item from genetic studies of common traits. We were able to effectively summarize GWAS results by taking advantage of numerous, small marker effects. Approaches such as ours, with the availability of genomic data, could be applied in the clinical setting to improve disease risk assessment, treatment, and prevention.

## Materials and Methods

### Study participants

The BHS is a long-term, longitudinal, cohort study investigating the natural progression of cardiovascular disease in children throughout the community of Bogalusa, Louisiana. Details outlining ascertainment have been previously described (Berenson and Pickoff, 1995). Briefly, cardiovascular disease risk factor examinations, including the collection of blood samples for lipid measurement, were performed on self-reported Caucasians and African Americans, at 3- to 4-year-intervals beginning from 1973 to 1974 and gathered through nine large cross-sectional surveys of children aged 4–17 years and 10 cross-sectional surveys of adults aged 18–48 years. This strategy has resulted in a panel of serial observations on individuals starting at childhood and extending through adulthood. Collectively, the derived data includes 12,163 individuals representing 37,317 unique observations. We focused on a subset of these individuals (*n* = 523), specifically unrelated Caucasians (238 male, 285 female) with between 4 and 13 unique observations per participant and available genotype data.

### Genotyping

Genotyping methods have been previously described (Smith et al., 2010). Briefly, genetic data on 1,202 BHS participants were obtained using the Illumina Human610 Genotyping BeadChip (Eberle et al., 2007) and HumanCVD BeadChip (Keating et al., 2008), with calls made using BeadStudio software. Quality control measures were implemented, resulting in 545,821 uniquely genotyped markers. The final average call rates were high (99.95 and 99.32%, respectively), with a high minimum call rate of the genotyped, known lipid genetic risk markers (99.15%), and high concordance between platforms (>99.98%) and high reproducibility (>99.99%). The degree of European ancestry for each study participant was calculated using a supervised clustering analysis (Libiger and Schork, 2012) from publicly available European reference panels (*n* = 1,335). Participants estimated to be less than 90% ancestrally European were removed from downstream analyses. Among remaining participants, relatedness was assessed using pairwise IBD estimation in PLINK (Purcell et al., 2007). Participants were excluded such that the estimated proportion of IBD between any two remaining individuals was less than 0.1.

### Imputation

Prior to imputation, genetic markers were excluded which demonstrated high missingness (>0.05), failed Hardy–Weinberg equilibrium (*p* < 0.0005), or had exceedingly rare alternative alleles (minor allele frequency <0.005). The remaining genetic data were pre-phased (Howie et al., 2012), and genome-wide imputation was performed on the resulting haplotypes using the default parameters in IMPUTE v2.2.2 (Howie et al., 2009). The 1000 Genomes Phase 1 integrated variant set haplotypes were used as the reference panel (Altshuler et al., 2010). Genomes were divided into approximately 5 Mb segments (avoiding chromosome and centromere boundaries) with phasing and imputation calculated on each. Imputed markers with information values less than 0.5 were removed from the analysis. GTOOL v0.7.0 was used to convert imputed genotyped posterior probabilities into calls. Genotypes were considered missing if the posterior probability of any genotype was not greater than 90%.

### Statistical analyses

The primary outcomes of interest were HDL-C, LDL-C, TC, and TG. In each analysis, these phenotypes were analyzed using various regression models controlling for age, sex, and body mass index as fixed effects covariates while treating the study participant as a random effect to account for the longitudinal nature of the data. All statistical analyses were performed in R v2.13.2 (R Development Core Team, 2011) using the packages *BLR* (de los Campos and Rodriguez, 2012) and *lme4* (Bates et al., 2011).

#### Modeling age

Lipid levels tend to display a sigmoidal trend over lifetime. We hypothesized that a function which accounts for this trend would be more appropriate than a linear term. However, as the present study includes longitudinal measurements, we chose to explore options which would still operate in a linear mixed model framework. For each observation *i*, we constructed the function *f*(age_i_), such that:

$$f\left(\text{ag}{\text{e}}_{i}\right)=\sin\left(\frac{\pi }{2}\cdot \frac{\text{2ag}{\text{e}}_{i}-\left(\text{ag}{\text{e}}_{\left(n\right)}+\text{ag}{\text{e}}_{\left(1\right)}\right)}{\text{ag}{\text{e}}_{\left(n\right)}-\text{ag}{\text{e}}_{\left(1\right)}}\right)$$

where age_(1)_ is the study minimum age and age_(*n*)_ is the study maximum age. Notably, this function achieves a maximum of one at the maximum observed age and a minimum of negative one at the minimum age. We compared model fit using AIC. In each case, the model using *f*(age*_i_*) had better fit (Table 4) and visually appeared more appropriate. Thus, we modeled age using *f*(age*_i_*) in all analyses.

**Table 4:** **Model fit statistics (AIC) using age as a linear term and the sine function, *f*(age_i_) in a non-genetic model**.

	Linear	Sine
HDL-C	32923.79	32923.58
LDL-C	36982.23	36916.02
TC	37922.29	37839.51
TG	40445.38	40428.51

#### Lipid genetic risk markers

Genetic markers that had been previously identified in GWAS as being associated with HDL-C, LDL-C, TC, or TG (*p* < 1.0 × 10^−7^), were derived from European populations, and present in the National Human Genome Research Institute (NHGRI) GWAS Catalog were included in the analyses (Dataset S1 in Supplementary Material). For each lipid measurement, two sets of markers were constructed: (1) all reported lipid markers (given the suffix A); and (2) only those genetic markers previously reported to be associated with that lipid (given the suffix R).

#### Non-genetic model

An *n*-fold cross-validation approach was used to assess predictive accuracy. One patient (and each of their observations) was first excluded. Lipid levels of the remaining study participants were regressed on fixed and random effect covariates. The corresponding fixed effects estimates were multiplied by the excluded patient’s fixed covariates and summed together to obtain a predicted lipid measurement. This method was repeated for each participant in the study, and the square of the Pearson’s correlation coefficient between the true and predicted outcomes was recorded. In mathematic terms, let **X***_i_* be a *k_i_* × *p* matrix where *k_i_* is the number of observations on the *i*th study participant where *i* = {1, 2,…, 523} and *p* is the number of fixed covariates. For the non-genetic model, *p* is equal to four (intercept, age, sex, body mass index). Similarly, let **X**_−*i*_ be a (*K* − *k_i_*) × *p* matrix where *K* is the total number of observations from all study participants, and each row corresponds to an observation in the dataset, excluding the *i*th study participant. Let **Z_−*i*_** be the study participant covariance matrix, and let **Y_−*i*_** be the vector of outcomes after excluding the *i*th study participant. Then the fixed effects estimate for *B_−*i*_*, ${\tilde{B}}_{-i}$, in the equation:

$$Y_{-i}=X_{-i}B_{-i}+Z_{-i}U_{-i}+\varepsilon_{-i}$$

can be calculated as ${\tilde{B}}_{-i}=X_{-i}^{\text{T}}R_{-i}^{-1}Y_{-i}$ where ε_−*i*_ is a vector of IID random error terms with mean zero and covariance matrix **R**_−*i*_ and *U*_−*i*_ is a vector of random effects. The predicted lipid levels for the *i*th study participant, ${\hat{\text{Y}}}_{i}$, are calculated as:

$${\hat{\text{Y}}}_{i}=X_{i}{\tilde{B}}_{-i}$$

This process is completed for *i* = {1, 2,…, 523} study participants. Finally, predictive accuracy, measured as the square of the Pearson’s correlation coefficient (and referred to as *R*^2^ in the text), between **Y** and $\hat{\text{Y}}$ is calculated, where **Y** = (**Y**_1_, **Y**_2_,…, **Y**_523_)^T^ and $\hat{\text{Y}}={\left({\hat{\text{Y}}}_{1},{\hat{\text{Y}}}_{2},\ldots ,{\hat{\text{Y}}}_{523}\right)}^{\text{T}}.$

#### NCBI risk score

The NCBI risk score was calculated using only markers previously reported to be associated with each lipid phenotype. It is identical to the weighted allele-counting method, where the reported estimates were assigned to each weighting factor. In instances where multiple studies reported association with the same marker, the estimate obtained by the largest study was used. Significance was assessed by regressing lipid levels on the risk scores, controlling for fixed and random effects. An *n*-fold cross-validation approach was then used to assess predictive accuracy after including the NCBI risk score. The method is equivalent to the method presented above, with the exception that *p* is equal to five; and in addition to intercept, age, sex, and body mass index, the **X** matrices include the NCBI risk scores.

In a similar manner, NCBI risk scores were assessed for age-specific association and predictive accuracy at 10-year-intervals between 10 and 40 years of age. Observations were included that were within 2.5 years of the desired age (e.g., 7.5–12.5 years of age for the age 10 interval). Only one observation per individual was included. In instances where multiple observations from the same interval occurred within the age range, the observation closest to the desired age was used. Additionally, at ages 20, 30, and 40, the NCBI risk model was assessed after including the most recent previous lipid measurement as a fixed effects covariate.

#### BHS risk scores

The BHS-A and BHS-R risk scores were calculated using all reported lipid risk markers and only those reported to be associated with the lipid measurement examined, respectively. Separate marker effects for each individual were calculated using *n*-fold cross-validation. These marker effects were subsequently incorporated into a weighted allele-counting procedure to generate a risk score (Figure 2). In mathematical terms, let **x***_i,j_* be the vector of (identical) genotypes for the *i*th study participant at the *j*th marker where *j* = {1, 2,…, *J*} and *J* is the number of markers in the set of interest. Similarly, let **x**_−*i*,*j*_ be the vector of genotypes for all others, excluding the *i*th study participant, at the *j*th marker. Then the marker effect estimate for β_−*i*,*j*_, ${\hat{\beta }}_{-i,j}$, in the equation:

$$Y_{-i}=x_{-i,j}\beta_{-i,j}+W_{-i}\theta_{-i}+Z_{-i}V_{-i}+e_{-i}$$

can be calculated as ${\hat{\beta }}_{-i,j}=x_{-i,j}^{\text{T}}S_{-i}^{-1}Y_{-i}$, where *e*_−*i*_ is a vector of IID random error terms with mean zero and covariance matrix **S**_−*i*_, **W**_−*i*_ is a matrix of fixed effects covariates with effects θ_−*i*_, and *V*_−*i*_ is a vector of random effects. The BHS risk score for the *i*th study participant, Φ*_i_*, can be calculated as:

$$\varphi_{i}=\overset{J}{\underset{j=1}{\sum }}x_{i,j}{\hat{\beta }}_{-i,j}$$

Lipid levels were regressed on the BHS risk scores to obtain significance measurements after controlling for fixed and random effects, and predictive accuracy was assessed in the same manner as presented above.

#### BLR and BRR models

The BLR and BRR models were fit using the *BLR* package (Howie et al., 2009) in R v2.13.2 (Howie et al., 2012), and predictive accuracy was assessed using *n*-fold cross-validation. Age, sex, and body mass index were treated as fixed effects covariates, and the study participant covariance matrix was used as the grouping factor. All markers in the set were included simultaneously using the lasso penalty in the BLR models and the ridge penalty in the BRR models. Posterior estimates of the fixed and marker effects from the training models were used to calculate the prediction values in the testing sets. Let *V*(*y*) be the variance of the lipid measurement in the training set, *h*^2^ = 0.5 be the heritability of the lipid measurement (Knoblauch et al., 1997), and *MS_x_* be the average sum of squares of the training genotypes. Then in the BLR model, priors were set to the following:

$$\begin{array}{ccc} \text{VarE}:df=5;S=V\left(y\right)\left(1-h^{2}\right)\left(df-2\right), & & \\ \text{lambda}:\text{type}=“\text{random}”;\text{value}=\frac{2\left(1-h^{2}\right)}{h^{2}}MS_{x}; & & \\ \text{shape}=2;\text{rate}=\frac{\left(\text{shape}-1\right)}{\text{valu}{\text{e}}^{2}}, & & \\ \text{VarU}:df=5;S=\frac{V\left(y\right)h^{2}}{3} & & \end{array}$$

And the BRR model priors were set as:

$$\begin{array}{c} \text{VarE}:df=5;S=V\left(y\right)\left(1-h^{2}\right)\left(df-2\right),\\ \text{VarBR}:df=5;S=\frac{V\left(y\right)h^{2}\left(df-2\right)}{MS_{x}},\\ \text{VarU}:df=5;S=\frac{V\left(y\right)h^{2}}{3} \end{array}$$

These values provide a prior expectation of the residual variance that is three quarters of the phenotypic variance and a relatively flat prior density over a wide range of the regularization parameter. When the degree of freedom is set to five, priors have finite mean and variance and a relatively small influence on inference (Makowsky et al., 2011). Let **X***_i_* be the *k_i_* × *m* matrix of genotypes for the *i*th study participant, where *m* is the total number of markers in the set, and let **W***_i_* be the matrix of fixed covariates. If $\beta_{-i}^{L}$ and $\beta_{-i}^{R}$ are the vectors of posterior genotype estimates for the BLR and BRR models, respectively; and $\theta_{-i}^{L}$ and $\theta_{-i}^{R}$ are the vectors of posterior fixed effects estimates for the BLR and BRR models, respectively, in the training models which exclude the *i*th study participant. Then the predicted outcomes for the *i*th study participant using the BLR model, ${\hat{\text{Y}}}_{i}^{L}$, and the BRR model ${\hat{\text{Y}}}_{i}^{R}$ are equal to:

$$\begin{array}{c} {\hat{\text{Y}}}_{i}^{L}=X_{i}\beta_{-i}^{L}+W_{i}\theta_{-i}^{L},\\ {\hat{\text{Y}}}_{i}^{R}=X_{i}\beta_{-i}^{R}+W_{i}\theta_{-i}^{R} \end{array}$$

The predictive accuracy of these models was calculated as described above.

## Supplementary Material

The Supplementary Material for this article can be found online at: http://www.frontiersin.org/Statistical_Genetics_and_Methodology/10.3389/fgene.2013.00086/abstract

Supplementary Dataset S1 **Genetic markers previously reported to be associated with lipid level phenotypes and previously reported parameter estimates**.

Supplementary Dataset S2 **Single marker association results**.

## Conflict of Interest Statement

The authors declare that the research was conducted in the absence of any commercial or financial relationships that could be construed as a potential conflict of interest.

## Footnotes

^1^http://www.genome.gov/gwastudies
